# Systematic assays and resources for the functional annotation of non-coding variants

**DOI:** 10.1515/medgen-2022-2161

**Published:** 2022-11-29

**Authors:** Martin Kircher, Kerstin U. Ludwig

**Affiliations:** Institute of Human Genetics, University of Lübeck, Lübeck, Germany; Berlin Institute of Health at Charité – Universitätsmedizin Berlin, Berlin, Germany; Institute of Human Genetics, University Hospital Bonn, University of Bonn, Venusberg-Campus 1, Building 76, 53127 Bonn, Germany

## Abstract

Identification of genetic variation in individual genomes is now a routine procedure in human genetic research and diagnostics. For many variants, however, insufficient evidence is available to establish a pathogenic effect, particularly for variants in non-coding regions. Furthermore, the sheer number of candidate variants renders testing in individual assays virtually impossible. While scalable approaches are being developed, the selection of methods and resources and the application of a given framework to a particular disease or trait remain major challenges. This limits the translation of results from both genome-wide association studies and genome sequencing. Here, we discuss computational and experimental approaches available for functional annotation of non-coding variation.

## Introduction

Over the past decade, technical capabilities to identify genetic alterations in individual genomes have increased substantially. However, two major challenges remain: (i) discriminating between pathogenic (causal) and benign variants; and (ii) understanding the effects of genetic variants at the molecular level. This is particularly true for the “non-coding” genome, which harbors both the majority of variants associated with common traits (as identified by genome-wide association studies [GWAS]) and an as yet unknown number of variants underlying monogenic disorders [1], [2].

**Figure 1:**
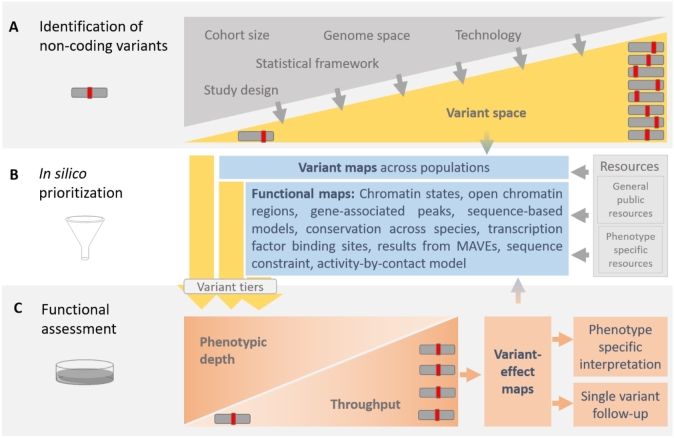
**Functional genomics of non-coding variants. (A) Defining the variant space.** Technological advances such as array- or sequencing-based methods have enabled the systematic identification of genetic variants in individual genomes. The variant space encompasses all identified candidate variants, for example, all variants observed in an individual patient in a clinical panel or exome, variants identified from a genome-sequenced case-parent trio, or those variants that meet a specific statistical threshold in cohort-based analyses (e. g., significance in genome-wide association studies). Thus, the size of this variant space is largely driven by the specific study design. **(B) Ranking of variants.** In situations where the variant space is small, these can be taken directly to functional assessment, or they can be ranked based on variant frequencies in different populations (variant tiers, yellow arrows). However, in most study designs, prioritization approaches are required. Here, functional maps (of experimental or computational origin) are integrated with the variant space to reduce numbers. Functional maps are drawn from publicly available web resources and databases, which can be general or specific to a certain phenotype. For a selection of resources, see Table 1. **(C) Mapping variant effects.** The experimental approach to investigate the functional effects of the prioritized variants is a trade-off between depth of molecular assessment and throughput (i. e., number of variants tested in parallel). Ideally, results of variant analyses are collected in variant-effect maps, which can then be used for interpretation in the phenotypic context and/or prioritize a limited number of variants for in-depth investigation, for instance in animal or organoid models. Importantly, variant-effect maps may inform prioritization in future studies of the same phenotype, if they are deposited in publicly available resources. Abbreviations: MAVE, multiplex analysis of variant effects.

To advance variant interpretation, large-scale collaborative efforts have generated extensive catalogs that document genetic variation and genomic elements, together with their respective molecular functions, across hundreds of cell types, including 3D genomic interactions [3]. Most of this information is deposited in public databases, which have emerged as knowledge hubs in human genomics. In addition, technological advances now provide the means to characterize the molecular effects of genetic variation at scale on an experimental basis. Nonetheless, the identification of appropriate resources and protocols, the assessment of relevant data, and the correct interpretation of experimental findings remains problematic [4], [5]. Here, we summarize how diverse experimental and computational approaches can be applied to advance interpretation of genetic variation in the non-coding genome (Figure 1).

## Resources for the non-coding genome

### Human genetic variation

Over the past decade, systematic global efforts have been made to catalog diverse types of genetic variants both across the allelic spectrum and across populations [3], [4], [6], [7]. These investigations have included the International HapMap Project, the 1000 Genomes Project, and the more recent Genome Aggregation Database (gnomAD) initiative. According to current estimates, each individual genome harbors 3 to 4 million small alterations of below 50 bp (the majority of which are single nucleotide variants [SNVs]), and around 15,000 structural variants (SVs, >50 bp) [8]. Additional variants will be identified as further progress is made towards completion of the human reference sequence (Telomere-to-Telomere (T2T) consortium; [9]).

Variant information is made accessible through web-based resources, which balance the issues of data sharing and privacy in order to benefit the medical genetics community (Table 1). The majority of variants are derived from observations in only one individual (“singletons”). Since some of these might represent either technical artifacts or disease-causing variants in unscreened individuals from population-based cohorts, researchers are encouraged to rely on cross-population allelic frequency, rather than on the mere presence of a variant. Importantly, variants identified from more than 100,000 individuals (across diverse populations) serve as a good basis to study sequence variation compatible with life [10]. The underrepresentation of variant alleles, or the clustering of trait-associated variants within a genomic region, can facilitate the prioritization of variants that are of functional relevance [11], [12], [13].

**Table 1:** **Important resources for the annotation of functional variants in the human genome.** Various online resources are listed in the categories “Genetic variation and associations,” “Large-scale functional genomics data sources,” “Browser and meta databases,” and “Enhancer, transcription factor, and element databases.” We tried to list the major resources, so this list must be incomplete and can only represent a limited view of available resources. Many of these websites provide data access through interactive searches and visualizations, while other sites serve as portals for data download and offline analysis.

Name	Description	URL
***Genetic variation and associations***		
BRAVO/TOPMed	BRAVO variant browser provides alleles, functional annotations, and allele frequencies from variants identified across genomes in the TOPMed project	https://bravo.sph.umich.edu/
gnomAD	Genome Aggregation Database providing aggregated and harmonized variants (incl. SVs) and their annotation from various large-scale exome and genome sequencing projects	https://gnomad.broadinstitute.org/
ClinVar	NCBI repository clinically annotated variant effects	https://www.ncbi.nlm.nih.gov/clinvar/
COSMIC	Catalogue of Somatic Mutations in Cancer and their annotations	https://cancer.sanger.ac.uk/cosmic/
GWAS Catalog	NHGRI-EBI Catalog of human genome-wide association studies, collecting region/variant associations from thousands of publications	https://www.ebi.ac.uk/gwas/
IGSR	The International Genome Sample Resource, incl. the Human Genome Diversity Project (HGDP) and the Simons Genome Diversity Project (SGDP)	https://www.internationalgenome.org/
1000 Genomes Project	International Genome Sample Resource of the 1000 Genomes Project providing links to individual level data from various populations	https://www.internationalgenome.org/
***Large-scale functional genomics data sources***		
ENCODE	Data portal of the Encyclopedia of DNA Elements, including for example TF and histone ChIP, open chromatin, and expression data	https://www.encodeproject.org/
EMBL-EBI Single cell atlas	Single-cell expression atlas across species, including the Human Cell Atlas	https://www.ebi.ac.uk/gxa/sc/home
FANTOM	Functional Annotation of the Mammalian Genome, including atlases of promoters, enhancers, long non-coding RNAs, and microRNAs	https://fantom.gsc.riken.jp/
GTEx	Genotype-Tissue Expression (GTEx) Portal with tissue-specific gene expression and regulation data	https://www.gtexportal.org/home/
HuBMAP	Human BioMolecular Atlas resource for discovery, visualization, and download of single-cell tissue data	https://portal.hubmapconsortium.org/
IHEC	Data portal of the International Human Epigenome Consortium incl. methylome, transcriptome, histone, and other data	https://ihec-epigenomes.org/
Roadmap Epigenomics	Integrative analysis of 111 reference human epigenomes	http://www.roadmapepigenomics.org/
pyschENCODE	Integrated resource of regulatory genomic elements in individuals with neuropsychiatric disorders	http://resource.psychencode.org/
4DN	4D Nucleome Network provides nuclear organization data as well as a platform to search, visualize, and download them	https://data.4dnucleome.org/
***Browser and meta databases***		
Ensembl Regulation	Ensembl Regulation provides computational annotation of regulatory features in the genome, incl. genome segmentation and annotation of regulatory features	http://www.ensembl.org/info/genome/funcgen/index.html
Gene Expression Omnibus (GEO)	NCBI repository for all kinds of functional genomics datasets and their structured metadata	https://www.ncbi.nlm.nih.gov/geo/
UCSC Genome Browser	Popular genome browser integrating data from various sources	https://genome.ucsc.edu/
WashU Epigenome Browser	Genome browser integrating epigenetic, 3D genome visualization, and image data	https://epigenomegateway.wustl.edu/
***Enhancer, transcription factor, and element databases***		
Altius Index	Human DHS index of about 3.6 million sites, providing a common coordinate system for regulatory DNA	https://index.altius.org/ (browser), https://www.meuleman.org/research/dhsindex/ (data)
ENCODE SCREEN	Registry of Candidate cis-Regulatory Elements from the ENCODE project	https://screen.encodeproject.org/
EnhancerAtlas	Experimentally derived enhancer annotation in nine species	http://www.enhanceratlas.org/
Gene Transcr. Regulation Db	GTRD provides uniformly processed ChIP-seq data for identification of transcription factor binding sites in human or mouse	https://gtrd.biouml.org/
GeneHancer	Genome-wide integration of enhancers and target genes in GeneCards	https://www.genecards.org/
JASPAR	Open-access database of transcription factor binding profiles	https://jaspar.genereg.net/
MaveDB	Open-source platform to distribute and interpret data from multiplex assays of variant effects (MAVEs)	https://www.mavedb.org
MPRAbase	Repository and uniform processing of massively parallel reporter assay (MPRA) datasets across several organisms	https://www.mprabase.com/
ORegAnno	Open resource for curated regulatory annotation, incl. about transcription factor binding sites, RNA binding sites, regulatory variants, haplotypes, and other regulatory elements	http://www.oreganno.org/
RegulomeDB	Annotation of SNVs with known and predicted regulatory elements in the intergenic regions of the human genome	https://regulomedb.org/
VISTA Enhancer Browser	Resource for experimentally validated human and mouse non-coding fragments with gene enhancer activity as assessed in transgenic mice	https://enhancer.lbl.gov/

Together with information from NCBI ClinVar or HGMD, “variant prioritization” based on allelic frequencies has been central to the identification of causal genes for many Mendelian syndromes [14]. However, few of the variants that are reported as causal in curated clinical databases are located outside of gene sequences, thus limiting the interpretation of variants identified in non-coding regions by genome sequencing. Furthermore, while public variant maps have facilitated the identification of common risk variants for multifactorial traits, functional interpretation is difficult, due to the fact that, again, most are located outside of protein-coding genes [15].

### Genomic architecture of the non-coding genome

By definition, the “non-coding genome” encompasses all of the sequence located outside of protein-coding elements (i. e., around 98 % of the human genome). It contains the majority of variants associated with common traits, as well as an as yet unknown number of causal variants for Mendelian diseases. Although widely used, the term “non-coding genome” does not reflect its true complexity, as illustrated by the wide diversity of molecular functions that have been associated with distinct sequence elements.

Non-coding elements can be located in close proximity to coding regions and are considered part of the respective genes. These elements include the 3′/5′ untranslated regions, the core promoter, and (deep) intronic splice regions, all of which have an established role in gene regulation [16]. In addition, “non-coding genes” provide the sequence for diverse RNA species, which are generally not translated into proteins (e. g., long non-coding RNA, microRNA). While they contribute to transcriptional and post-transcriptional regulation of their target genes, the map of non-coding genes remains incomplete [17].

However, most regulatory sequence elements are located outside of genic regions and are difficult to predict from sequence alone. Regulatory sequence elements are composed of: (i) “proximal regulatory elements,” which are located close to transcription start sites; and (ii) “distal regulatory elements,” which are located further away. Both are in contact with their target genes through spatial interaction and, based on different resources (Table 1), they cover an estimated 5–20 % of the genome. Their interactions occur predominantly within the context of regulatory units [18], with topologically associating domains (TADs) representing the basic domains of the 3D genome architecture [19]. Importantly, the activity state of a specific regulatory element (e. g., an active “enhancer” and a repressive “silencer”) is largely dependent on the presence of cell type-specific binding proteins (e. g., transcription factors), and can vary between cell types.

### Functional maps of the “non-coding” genome

In contrast to the technical ease of identifying human variation at the individual and population levels, current capabilities for understanding the functional effects of non-coding variants at the molecular, cellular, organismal, and ultimately phenotypic level remain limited. This is largely attributable to our limited understanding of sequence elements in the non-coding genome, which is mainly due to: (i) the lack of a universal translation code comparable to the amino acid sequence in protein-coding genes; (ii) the temporal and spatial activity of regulatory elements complicating their study; (iii) limited understanding of general gene regulation processes; and (iv) the presumably small, but as yet unknown, effect sizes of most non-coding variants.

To improve understanding of the non-coding genome, functionally relevant genomic elements must first be cataloged and annotated in a systematic manner [5]. Here, “functionally relevant” refers to sequence blocks of variable length that contribute to the spatio-temporal expression of genes, hence the term “regulatory.” The Encyclopedia of DNA Elements (ENCODE) represents the first attempt towards the global mapping of sequence elements that are indicative of regulatory activity (i. e., DNA accessibility, histone modifications, methylation patterns), across diverse human tissues and developmental stages. Additional projects, such as the EU BLUEPRINT Epigenome initiative, the International Human Epigenome Consortium (IHEC), NIH RoadMap Epigenomics, and the Functional Annotation of the Mouse/Mammalian Genome (FANTOM) consortium (Table 1), have further advanced the “functional genome map” by providing additional tissues and assay types (e. g., immunoprecipitation of DNA binding proteins [transcription factors and histones], 3D organization, and interaction of DNA elements).

Importantly, these datasets are often enriched for specific tissues and cell types, with a major bias towards those that are easily obtainable (e. g., immune cell types from blood). In addition, funding for specific projects has often been provided within the context of specific research areas (e. g., the focus of the BLUEPRINT Epigenome is the hematopoietic system). Therefore, the use of public data for prioritization approaches may be hampered by inherent biases in data acquisition, and thus a lack of general applicability.

Functional maps are often accessible through the graphical interfaces of web-based portals (Table 1). In contrast to the situation for genetic variation, here, data privacy is only of limited concern, and both unprocessed and processed data are often made available. Although a large number of molecular assays are performed, each provides only one functional dimension, and integrated approaches are often required to provide functional annotation (e. g., diverse histone modifications combined into chromatin segments [20]).

Visualization of functional maps is most helpful when used for specific chromosomal regions (e. g., a GWAS risk locus) and/or when a disease-relevant cell type is already known (and available). If this is not the case, functional maps can be used to identify the disease-relevant cell types by calculating enrichments of non-coding variants in regulatory elements. To enable such systematic analyses, summary level data on the entirety of regulatory elements across different cells and tissues can be downloaded (e. g., from the SCREEN database [21] or Ensembl Regulatory Build [22]) and used for enrichment approaches. Methods for enrichment analyses have been excellently reviewed elsewhere [23].

### Integration of genotypes and (molecular) phenotypes

Individual projects have also analyzed primary tissues and embryonic cell types, as well as cells derived from non-European populations, since the influence of population background on cell type-specific gene regulation remains unclear. While these studies complete the existing functional maps, they often lack the resources required to maintain web portals and thus release their data in general databases such as NCBI Gene Expression Omnibus (GEO). Tens of thousands of functional genomics datasets are available in GEO, including data from diverse model organisms, which enable interspecies analyses as promising orthogonal avenues [24], [25]. The widespread availability of functional maps in public domains such as ENCODE and GEO provides enormous potential for advancing the interpretation of non-coding risk variants. However, one current challenge in this respect is the requirement for the uniform reprocessing of data when multiple studies are combined.

To facilitate the investigation of the impact of common genetic variants on molecular functions, the Genotype-Tissue-Expression (GTEx, [26]) Project was established in order to generate maps of quantitative trait loci (QTLs), in which genotypes are statistically correlated with molecular measures at the population level. Following their application to investigate gene expression in bulk (i. e., expression QTLs [eQTLs]), QTL studies have since been extended towards the investigation of a variety of molecular functions, such as splicing (sQTLs), methylation (meQTL), chromatin accessibility (caQTL), and even the regulation of protein abundance (pQTL). Importantly, the observed correspondence between genotype and molecular measure represents only a statistical correlation between two traits, and orthogonal evidence is required to delineate the biological mechanism. The latter might include Bayesian approaches, such as colocalization, or experimental manipulation [23].

### Beyond single nucleotide variants

SNVs are the most abundant form of genetic variation and are the most comprehensively cataloged to date due to the technical ease of their identification. Unsurprisingly, therefore, most annotation efforts for non-coding variants focus on SNVs. However, variant types that encompass a larger number of nucleotides, such as SVs, are probably more powerful in terms of causing functional effects of regulatory elements. For instance, whereas the absence of an entire transcription factor binding motif is more likely to abolish binding, an SNV within the motif is likely to modify binding affinity in a quantitative manner. Studies of patient-specific SVs have already suggested disease-causing mechanisms in non-coding regions, particularly within the context of TADs [19], [27]. Since the identification of SVs is becoming ever easier due to technological and algorithmic advances (e. g., long-read sequencing, optical mapping) [8], an increasing number of high-quality SVs is anticipated. While these provide the opportunity to study regulatory effects, interpretation might also become more complex, as SVs are likely to harbor multiple genomic elements simultaneously, each of which might contribute to a different molecular function [28], [29].

## Prioritization approaches

Intuitively, the number of candidate non-coding variants identified by large-scale GWAS might be larger than those identified by studies on *de novo* mutations (DNMs) in rare diseases. However, the exact number of candidate variants (i. e., the “variant space”) is largely influenced by study design, sample size, and the genetic architecture of the trait (Figure 1). For example, an analysis of genome-wide data from 1,000 trios would result in around 80,000 candidate DNMs, whereas GWAS for traits with limited biological complexity (e. g., orofacial clefting) have identified a few dozen risk loci with a few hundred candidate variants to date. The aim of *in silico* prioritization approaches is to provide a relative ranking of candidate variants, thereby allowing a reduction in the number of variants forwarded for experimental follow-up.

### Non-coding *in silico* scores

Two major types of *in silico* scores are currently available, i. e., “specialized scores” and “broadly applicable scores.” Specialized scores assess the impact of a variant on specific molecular functions and are particularly powerful for candidate variants with *a priori* functional hypotheses, e. g., those located in splice regions, within binding sites of transcription factors or miRNAs, or in regions of open chromatin. In contrast, “broadly applicable scores” make use of general annotations such as sequence constraint, i. e., conservation across species, or metrics derived from variant density at a certain region. Sequence constraint is a particularly powerful measure, as it integrates diverse molecular effects through organismal fitness and survival, at the cost of not necessarily providing a base pair resolution. This concept is commonly used in the context of annotating deleterious variants in protein-coding regions (e. g., missense Z-score in gnomAD), and can be readily transferred to non-coding regions. Nonetheless, at writing, no single score from either group is an effective predictor across all variant types. To improve predictions, multiple scores can be integrated into one measure of deleteriousness [30]. Examples of tools that use genomically broad (and less biased) datasets include Eigen, LINSIGHT, and CADD.

Again, the generalizability of *in silico* scores is limited by the available data. Particularly in the case of scores that are derived from specific experimental assays via machine learning, the predictive power is limited to those functions that are represented in the training data. For instance, if no feature covers the effect of a cell type-specific transcription factor, the resulting score will not be predictive of such molecular effects when annotating candidate variants. Furthermore, *in silico* scores that rely on conservation may fail in the prediction of “gain-of-function” variants, e. g., the generation of new transcription factor binding sites. It was previously demonstrated that results from experimental data of regulatory variant effects (e. g., from multiplex assays of variant effects [MAVEs], see below) are not well captured by any of the existing *in silico* scores [31].

To overcome these limitations, novel computational methods must be developed [31], [32]. Currently, the best results are obtained from sequence models that learn active and inactive motif representations from large collections of open chromatin data and histone marks (e. g., DeepBind [33], gkmSVM [34], DeepSEA [35], and Enformer [36]). These sequence models are publicly available and can be applied to variants of interest, although they may be biased by the representation of cell and tissue types in the respective training data. Model specialization (“transfer learning”) on matching cell type data might reduce such effects, and this is an area of active development within the computational field.

### Layered prioritization approaches

A wide range of computational pipelines are available, thus creating a plethora of prioritization options for any given list of variants. Therefore, consecutive (“layered”) approaches have become popular. These include: (i) considering variants with a specific predicted molecular effect only; (ii) removing variants above a certain allele frequency; (iii) applying the requirement for colocalization with certain histone modifications or open chromatin annotation; and (iv) filtering for conservation. Importantly, each of these layers reinforces the applied assumptions (e. g., inverse correlation of allele frequency and effect size), despite our still incomplete biological understanding of, and substantial evidence for exceptions to, all these proposed rules. To enable a more systematic characterization, the effects of the individual prioritization layers must be investigated one criterion at a time with the inclusion of a random set of all variants, or using a fully crossed design. Ideally, a compendium of scalable assays, each addressing a certain aspect of molecular read-out, should also be available. MAVEs (see below) and advances in synthetic biology represent initial steps in this direction.

### Approaches to link non-coding elements to target genes

Regulatory sequence elements can be located upstream or downstream – or even on a different chromosome [37] – than its target gene(s). This renders the assignment of links between genes and regulatory elements (gene-to-regulatory element link [GRL]) difficult, which is often required for the performance of gene set analyses and the generation of biological hypotheses. The most commonly used approaches to assign GRLs are on the basis of proximity, considering either the closest gene or both neighboring genes (potentially within certain distance limits). Alternatively, all genes within certain genomic windows, or all genes within TADs, are considered target genes. GRLs may also be inferred from experimental data, e. g., from diverse chromatin capture datasets; coexpression/coactivity data obtained from matched open chromatin and expression data across multiple cell-types; or the Activity-By-Contact (ABC) model, which considers both chromatin interaction and accessibility/histone acetylation data [38]. Once GRLs are established, a wide variety of gene annotation-based methods can be applied, ranging from gene set analyses to pathway and network enrichments.

## Multiplex assays of variant effects

The necessity for experimental testing of thousands of variants, coupled with advances in next-generation sequencing (NGS), has driven the development of MAVEs [39] and the collection of their results in an open repository, MAVEdb [40]. At their core, MAVEs allow systematic screening of variants in a single quantitative experiment and are primarily intended to identify variants with the potential for a specific molecular effect. We describe two major types of MAVEs for non-coding regions below. Importantly, the results of MAVEs typically require subsequent validation in an organismal system, often including organoid or animal models [41].

### Massively parallel reporter assays

**Figure 2:**
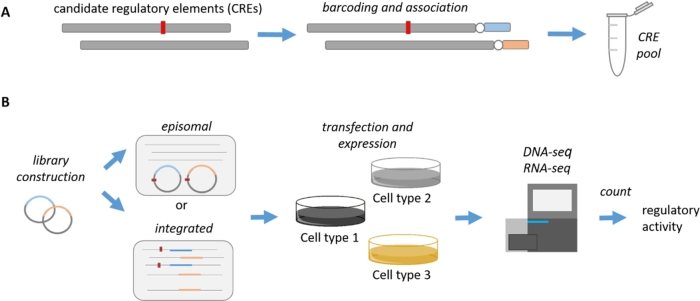
**Principle of massively parallel reporter assays (MPRAs).** MPRAs are used to simultaneously test hundreds to thousands of variants for potential regulatory effects in one assay. **(A) Generation of MPRA pools.** First, the genomic sequence around each variant (candidate regulatory elements [CREs]) is synthesized or otherwise derived and then combined with an individual barcode. All barcoded sequences are combined into one pool. **(B) MPRA reporter assay.** This pool is then cloned into vectors. The vectors contain a reporter gene (potentially driven by a minimal promoter) and place the CRE upstream of the transcriptional start site, while the barcode becomes part of the transcript’s 3′ or 5′ untranslated region. Depending on the assay type, the vectors are designed to either remain episomal or integrate into the genome (e. g., by lentivirus). The vector pool is then transfected (or transduced) into cell types of interest where the reporter genes are expressed. Following extraction of DNA and RNA from those cells, barcodes can be converted into highly complex sequencing libraries and read out on a high-throughput sequencing device. A regulatory effect of a certain CRE can be inferred from the number of detected barcode sequences at the RNA level, corrected by the number of transfected plasmids (detected by the barcode abundance in DNA). Allelic effects are derived from comparing the inferred expression effect of CREs with and without the allele of interest.

Massively parallel reporter assays (MPRAs, alternatively CRE-seq or STARR-seq) test the capability of putative regulatory elements to trigger gene expression in a specific cellular context. In MPRAs, thousands of short sequences (typically 150–300 bp) containing the regions (or variants) of interest are first created by oligo synthesis, which is the method of choice for the assessment of many independent variants, e. g., those located across loci. Alternatively, existing variation in cell lines can be utilized [42], [43], or error-prone PCR (”saturation mutagenesis”) can create all possible SNVs within a region of interest. The oligo-pool containing all candidate sequences is cloned into plasmid vectors, which are subsequently introduced into an *in vitro* system (Figure 2). Here, the plasmids either remain episomal, or are integrated into genomes through a lentiviral or other system [44]. The latter has been proven to be more powerful for cell types that are difficult to infect (e. g., neurons) or when a nucleosome context is required for read-out. To minimize the large positional effects of random integration [45], flanking insulators to the vector can be included [46].

Regulatory effects are assessed (or “read out”) by either quantification of the relative abundance of individual reporter RNAs by NGS compared to their DNA abundance, or a molecular phenotype (e. g., cell proliferation/death, fluorescence of the reporter), and can be correlated with specific variants within the tested element. To date, large MPRA datasets of regulatory variant effects (“variant-effect maps”) have been created in specific cell types, for specific loci [31], and for common variants identified by QTL studies or GWAS [42], [43], [47]. In a recent study, MPRAs were applied in clinical research to implicate differing transcriptional networks in two phenotypically similar neurodegenerative disorders [48].

**Figure 3:**
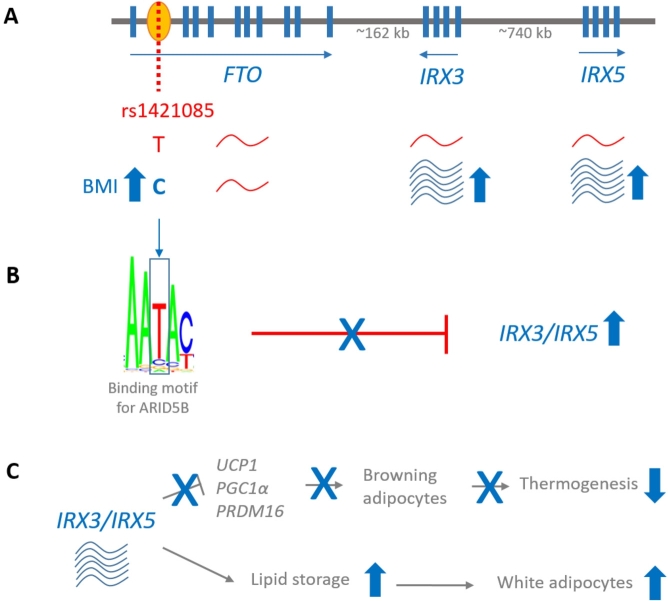
**Functional dissection of the** ***FTO*** **locus and its role in determining body mass index (BMI). (A) Genetic locus.** Genome-wide association studies have revealed an association between a region on chromosome 16q12 and BMI. Three genes are located within a 1-Mb genomic interval. One of the associated non-coding SNVs, rs1421085 (red line), is located within a regulatory sequence (yellow oval). Correlating individual genotypes and expression levels in adipocytes revealed that the C-allele is associated with increased expression of the genes *IRX3* and *IRX5*, while no genotype-dependent effect on *FTO* expression was observed. **(B) Molecular pathway leading to** ***IRX3*****/*****IRX5*** **overexpression.** The C-allele disrupts the binding motif of the transcription factor ARID5B, which is a repressor of *IRX3*/5 expression. In the presence of the C-allele, ARID5B does not bind, and the expression levels of *IRX3* and *IRX5* increase. This pathway explains the observed correlation between the C-allele and *IRX3*/5 expression as depicted in panel (A). **(C) Cellular mechanism.** In pre-adipocytes, IRX3 and IRX5 influence the cellular fate of pre-adipocytes; these can either develop into brown adipocytes, which contribute to energy consumption through thermogenesis, or white adipocytes, in which energy is stored through lipid accumulation. In the presence of an increased amount of *IRX3*/5, there is an increased number of cells shifting to the white adipocyte trajectory, while few brown adipocytes are generated. This results in reduced energy consumption in the presence of increased lipid storage.

### CRISPR/Cas9 approaches

The introduction of CRISPR/Cas9 paved the way for the development of in-genome MAVEs that retain the original local genomic context. In the first study to apply CRISPR/Cas9 genome editing in the context of MAVEs, Findlay et al. generated all possible SNVs in exon 18 of *BRCA1* [49], and used effects on nonsense-mediated decay, exonic splicing, and cellular growth as read-out. This application to coding regions influenced the way in which the non-coding genome is investigated by CRISPR/Cas9 genome editing [50], and recent developments include the integration of single-cell technologies for functional read-out [51]. Based on its capabilities to create highly specific sequence alterations, future applications of the CRISPR prime editing system are anticipated to replace other systems for sequence perturbations [52]. In combination with multiplexed read-outs, this may ultimately replace current vector-based MPRA studies.

CRISPR-based alternatives to genome editing include different CRISPR activation (CRISPRa) and interference (CRISPRi) screens [53]. Here, a specific locus or allele is targeted by a modified CRISPR fusion protein which no longer introduces strand breaks (e. g., dCas9 fusions), but instead serves as a sequence-specific probe. This probe tows an epigenetic modifier – which either increases or impairs gene expression – to a region of interest. With (single-cell) RNA-seq as the molecular read-out and combinatorial CRISPR targeting, the functional impact of candidate loci or allelic variants can be explored within regulatory networks [54].

### Limitations of MAVEs

MAVEs share the same limitations as low-throughput functional assays. First, they are performed in individual cellular systems, which require *a priori* knowledge regarding the most appropriate cell type for the trait of interest. Second, the results and interpretation are specific to the applied cell type [55], [56], [57], and do not capture organismal effects that might originate from the interaction of various cell types. Third, even if the relevant cell type(s) are known, the respective cell models might be unavailable and can only be replaced in part, e. g., by immortalized cell lines, since the applied alternatives do not capture the true biological identity in its entirety. Performing MAVEs that test effects across a number of cell types and/or conditions might generate the most robust results.

To maximize the biological insights provided by MAVEs, certain technical aspects also require further improvement. First, complementary high-resolution read-outs at the molecular and cellular levels are required to measure phenotypes at scale. This is particularly relevant for the phenotypic effects of the more common alleles, which are likely to be subtle for broader phenotypes, but will become detectable with more precise molecular read-outs. Second, current technical restrictions in DNA synthesis technology limit DNA fragment sizes, while for longer fragments, the capacity of the plasmid vectors imposes an artificial size limit. Here, novel approaches in synthetic biology that enable the analysis of larger fragments would provide superior coverage of the broad size range of regulatory elements, including the assessment of 3D interactions.

## From GWAS to molecular mechanism: The *FTO* locus in obesity

Early GWAS identified an extended haplotype block of 89 common variants, located in introns 1 and 2 of a gene named *Fat Mass And Obesity Associated* (*FTO*), as a risk locus for obesity (as measured by high body mass index [BMI]). *FTO* encodes a protein involved in the oxidative demethylation of various RNA species, and thereby contributes to post-transcriptional gene regulation. The absence of any deleterious coding variants suggested a regulatory effect of the risk haplotype, and *FTO* was considered the major positional candidate gene based on functional evidence from mice [58].

In a seminal study, the causative molecular pathway was identified via functional genomics ([59]; Figure 3). First, the authors intersected the regional association statistics with chromatin state annotations using 127 samples from the Roadmap Epigenomics project (see Table 1). A putative enhancer region of 12.8 kb in size was identified and found to be active in adipocytes. The integration of expression data suggested a key role for pre-adipocytes. These represent a specific adipocyte type, which develops along one of two trajectories into either (i) white adipocytes, i. e., fat-storing cells, or (ii) beige/brown adipocytes, which contribute to fat consumption via heat generation (“thermogenesis”). Integration of eQTL and chromatin interaction data from adipocytes suggested the genes *Iroquois Homeobox 3* (*IRX3*) and *IRX5* – both of which are master regulators of thermogenesis – as the enhancer’s targets. Having established both the implicated cell type and the regulatory targets, the causal variant was then determined via analysis of sequence conservation, transcription factor binding motifs, and gene coexpression. The analyses highlighted one single SNV (rs1421085) from the long haplotype block, involving a T-to-C transition within the region encoding the binding motif of the transcriptional repressor ARID5B.

Ultimately, the authors presented robust evidence that in the presence of the C-allele of rs1421085, ARID5B cannot bind to its target motif within the enhancer region. This results in increased expression of *IRX3*/*IRX5* in pre-adipocytes, which are then prompted to shift their developmental trajectory towards white adipocytes. This results in increased lipid storage and a simultaneous reduction in fat consumption via thermogenesis in beige adipocytes. This study reinforces the earlier notion that *a priori* reliance on the “nearest” gene might misguide functional follow-up.

## Concluding remarks

The interpretation of variants in the non-coding genome is a key challenge in the path towards personalized medicine. While researchers now appreciate the diversity of the molecular functions of non-coding elements, our knowledge of the full extent of regulatory principles and their complex interactions remains incomplete. In addition, currently available data are restricted to specific cell types (developmental stages, cellular conditions) and molecular assays, which limits efforts to predict variant effects.

In situations in which genomic datasets from different labs must be combined, joint analyses are complicated by cross-study differences in experimental and computational pipelines. In this respect, the importance of large-scale consortia such as ENCODE or gnomAD should be emphasized. These provide standards for experimental protocols, reagents, and terminology, and have pioneered data accessibility via the initiation of data portals with versioned and uniform data processing pipelines, genome browsers, and/or application programming interfaces (APIs), which enable scripted access and data download. However, the maintenance and sustainability of these databases is problematic due to the time-limited and project-specific nature of the respective funding periods.

Over the next decade, a major aim of research will be the efficient engineering of genomic alterations in order to assess their functional read-out in biological systems. Due to the technical challenges associated with MAVEs, the routine performance of these high-throughput approaches across multiple laboratories is unlikely. A more plausible scenario is that specialized academic centers will perform these analyses for a particular region of the genome or a disease of interest, and that the generated data will then be made available to the wider research community. However, to perform these experiments at scale and to enable sustained data accessibility, substantial funding will be required. An initial effort towards this goal is the recent foundation of the Impact of Genomic Variation on Function (IGVF) Consortium [60]. This was established in order to evaluate the function and phenotypic outcomes of coding and non-coding genomic variation using currently available approaches, and to develop improved experimental and computational strategies.

The coming years will see an enormous expansion in functional genomics datasets at all levels, i. e., with respect to novel experimental read-outs, additional annotations, and a variety of computational tools including scores, analysis pipelines, and machine learning approaches. While this opens up substantial opportunities for the field, the enormous challenges associated with data aggregation will complicate the use of these resources by the research community. A specific aim of consortia such as IGVF is to also facilitate data access by establishing variant-to-effect catalogs, including options to visualize variant impacts within the context of the underlying data, tools, and models. Together with additional key players, such as the Global Alliance for Genomics and Health (GA4GH) and European infrastructure projects such as ELIXIR, joined efforts must be established to build global resources for the interpretation of the non-coding genome. This will be required to introduce precision medicine across the broad medical genetics community.
